# Puppies in the problem-solving paradigm: quick males and social females

**DOI:** 10.1007/s10071-022-01714-5

**Published:** 2022-11-22

**Authors:** Claudia Pinelli, Anna Scandurra, Alfredo Di Lucrezia, Massimo Aria, Gün R. Semin, Biagio D’Aniello

**Affiliations:** 1 Department of Environmental, Biological and Pharmaceutical Sciences and Technologies, University of Campania “Luigi Vanvitelli”, Caserta, Italy; 2 Department of Biology, University of Naples Federico II, Via Cinthia, 80126 Naples, Italy; 3 Department of Economics and Statistics, University of Naples Federico II, Via Cinthia, 80126 Naples, Italy; 4 William James Center for Research, ISPA-Instituto Universitario, 1149‑041 Lisbon, Portugal; 5 Faculty of Social and Behavioral Sciences, Utrecht University, Utrecht, The Netherlands

**Keywords:** Animal cognition, Dogs, Problem-solving, Puppies, Sex differences

## Abstract

We report an observational, double-blind study that examined puppies’ behaviors while engaged in solving an experimental food retrieval task (food retrieval task instrument: FRTI). The experimental setting included passive social distractors (i.e., the dog’s owner and a stranger). The focus was on how the social and physical environment shapes puppies’ behaviors according to sex. The dependent variables were the number of tasks solved on an apparatus (Performance Index) and the time required to solve the first task (Speed). Sex and Stress were set as explanatory factors, and Social Interest, FRTI interactions, other behavior, and age as covariates. The main findings were that male puppies solved the first task faster than females. On the other hand, females displayed significantly more social interest and did so more rapidly than males. Males showed delayed task resolution. This study demonstrates sex differences in a problem-solving task in dog puppies for the first time, thus highlighting that sexually dimorphic behavioral differences in problem-solving strategies develop early on during ontogenesis.

**Supplementary Information:**

The online version contains supplementary material available at 10.1007/s10071-022-01714-5.

## Introduction

Sex differences depend on factors differentially affecting the reproductive success of the sexes. Females’ reproductive success is influenced by the production and care of offspring, whereas males’ fitness is directly proportional to the number of females inseminated (Bateman 1948; Fitzpatrick et al. 1995; Hood-Williams 1996; Rosvall 2011; Rubenstein and Lovette 2009; Shuster and Wade 2003). Therefore, sex-specific traits and abilities could be selected and maintained by sexual selection as an effect of intra-sexual competition and mate choice (Schuett et al. 2010).

In recent decades, research into the cognitive processes of dogs and their behaviors has steadily increased, and sex differences in behaviors not directly linked to reproduction have been an important area of interest (e.g., Aria et al. 2021). Of note, dogs have retained sex differences observed in wild species despite artificial selection (Scandurra et al. 2018a). For instance, female dogs appear more sociable toward humans (Lore and Eisenberg 1986; Wilsson and Sundgren 1997) and are more likely to display cooperative behaviors than males while solving a task (Foyer et al. 2013; Hori et al. 2013; Junttila et al. 2021; Persson et al. 2015). Female dogs are more responsive to visual signals than males, irrespective of whether they are social signals (D’Aniello et al. 2016) or physical signals (Müller et al. 2011). Behavioral responses to social olfactory stimuli (D’Aniello et al. 2021; Hamilton and Vonk 2015) and environmentally produced stimuli (Siniscalchi et al. 2011) have been sexually dimorphic. Sex differences have also been observed as a function of task type. These can be socio-cognitive tasks where a dog’s success depends on its ability to process, understand, and use the behaviors of another subject. Thus, in this task, a dog has to solve a problem after watching a ‘demonstrator’ (Pongrácz et al. 2004; Range et al. 2009; Scandurra et al. 2016; Topál et al. 2006).

In contrast, in the case of physical-cognitive tasks, the dogs have to solve the problem autonomously. An example is orientation tasks (T-maze paradigm), where female dogs learned better than males (Mongillo et al. 2017), while males appeared to be better at changing their navigation strategies (Fugazza et al. 2017). Moreover, the ability to switch from egocentric to allocentric (and vice versa) navigation strategies decreased with age in male dogs while increasing in females (Scandurra et al. 2018a). Duranton et al. (2015) studied sex differences in a physical problem-solving task where adult dogs had to open a box to retrieve food treats. They found that males were faster than females in the first trial, but females outperformed males in subsequent trials. The male advantage in the first trial was, according to them, due to their boldness (Goddard and Beilharz 1982; Svartberg 2002), which meant that they handled the task better as well as their lower level of neophobia (Goddard and Beilharz 1982). On the other hand, the subsequent superiority of females was explained by their ability to remember the successful problem-solving strategies applied in the previous tasks and possibly a reduction in their neophobia (Duranton et al. 2015).

Despite several adult studies, puppies’ problem-solving behaviors have not attracted much interest. At the same time, such research could be informative about the potential reasons underpinning sex differences, since puppies have not yet reached sexual maturity. Some sex differences have been reported for puppies (Wilsson and Sundgren 1998). For instance, in assessing the predictive effectiveness of puppy tests for adult behavior, it was found that female puppies were more active and independent than males. In contrast, male puppies were more dominant than females in competitive tendencies tests (Scott and Fuller 1965). Lazarowski et al. (2021) tested puppies of different ages for selection as detection dogs in three different testing procedures: Performance Test, for measuring the searching abilities and reward engagement; Emotional Reactivity Test, for evaluating the behavioral responses after provocative stimuli; Environmental Test, for studying puppies in a natural scenario. Overall, although male puppies scored worse than female ones on arousal, they performed better on several measures, such as working proneness and reward motivation.

Two studies on the impossible task paradigm failed to report sex differences in puppies (Lazarowski et al. 2019; Passalacqua et al. 2011). However, the latter two studies were not designed explicitly for detecting sex differences. Indeed, Lazarowski et al. (2019) did not control for sex. Therefore, the current study examined the performance of puppies on a physical problem-solving task according to sex. Since adult females appeared more engaged in social behaviors than males in problem-solving tasks (Foyer et al. 2013; Hori et al. 2013; Junttila et al. 2021; Persson et al. 2015), we also introduced passive social distractors during the test by including the owner and a stranger in the experimental room. This allowed us to examine the puppies’ interactions with people as a sign of their social interest, potentially affecting the outcome. Duranton et al.’s (2015) study suggests that the lower level of neophobia favors males’ confidence in novel tasks and thus higher success.

Sex is a dimorphic trait since adult males and females have different roles and behavioral ecology. Puppies do not show different roles and a different behavioral ecology; thus, we should hypothesize no sex-based behavioral differences. However, since the papers cited above (Lazarowski et al. 2021; Scott and Fuller 1965; Wilsson and Sundgren 1998) report sex differences already present in puppies, we refrained from formulating predictive hypotheses.

## Material and methods

We evaluated 77 domestic dog puppies, 3–6 months old, during the third development period, namely the “juvenile’’ period (Battaglia 2009). The sample consisted of not neutered dogs belonging to mixed breeds and mongrels (38 females, age ± SD = 4.79 ± 1.07; 39 males, 4.77 ± 0.99 months). All puppies lived in a human household as pets with at least two people at the testing time. The puppies were recruited by their owners through personal contacts and the Internet. Three puppies were not tested, since they showed no substantial interest in the food.

The puppies were tested in a standardized environment at the University of Naples Federico II. The test room was about 12 m^2^ and unfamiliar to the dogs. All the tests were video recorded with a closed-circuit television system and four cameras in the room’s corners.

Before the trial, the owners were informed about the testing procedure without explaining the study’s goal. The puppy–human pairs were then moved to the experimental area where a person unknown to the puppy was already present and took the role of the stranger. The two people (stranger and owner) were positioned with about a meter’s distance between them.

**Fig. 1:**
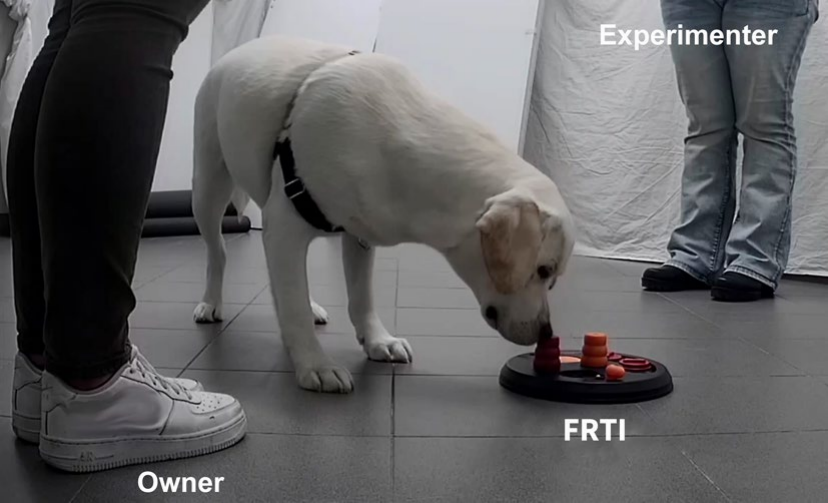
Screenshot of the experimental setup. In the test room, the two people [owner and experimenter (i.e., stranger)] were positioned at about a meter’s distance. The puppies had to retrieve treats by manipulating a food retrieval task instrument (FRTI)

The test consisted of a single 2-min trial per puppy whereby the puppies had to retrieve treats by manipulating a food retrieval task instrument (FRTI), a Flip Board Strategy Game (Trixie) they had never experienced before. This board game, projected for small dogs, consists of cones and indentations with hinged lids and sliders for a total of six tasks to be solved to retrieve food. The puppies could solve five tasks, since one of the hinges was broken. An experimenter placed the FRTI between the stranger and the owner (Fig. 1).

During the test, both the owner and the stranger remained inactive even if they were solicited by puppies, with a constant gaze not directed at either the FRTI or the puppy. At the beginning of the trial, the owner held the dog by the collar, while the experimenter settled the FRTI. As soon as the experimenter left the testing area, the dog was released and was free to approach the FRTI. After each test, the FRTI was washed with a non-toxic disinfectant.

We measured the number of tasks solved (hereafter Performance Index) by the puppies from 0 to 5 (depending on the number of tasks solved on the FRTI). Additionally, the performance latency to solve the first task was measured (hereafter labeled Speed), irrespective of which one.

The frequency and latency of any action directed toward people or the FRTI (i.e., visual, tactile approach, and go to the target) were considered. Most behaviors were not systematically displayed in our samples, giving sparse matrices inflated toward zero values, making the statistical analysis unreliable. Therefore, we grouped all single behaviors sharing the same goal to obtain denser matrices (see Table 1). Accordingly, all the FRTI-directed behaviors indicating the willingness to solve the tasks were added, and the variable was named “FRTI Interactions”. Similarly, all the owner- and stranger-directed behaviors were grouped in the variable named “Social Interest” after controlling that there were no differences between the behaviors directed to the owner and the stranger by non-parametric tests (females: Wilcoxon Signed Ranks Test frequency: *z* = − 1.06, *p* = 0.29; latency: *z* = − 0.23, *p* = 0.82; males: frequency: *z* = − 0.95, *p* = 0.34; latency: *z* = − 0.52, *p* = 0.60). Likewise, stress signals were added, such as yawning, scratching, shaking, licking lips, barking, and locomotion without a clear target. However, many dogs (73%) did not manifest any stress signals. This outcome would contribute to the limited frequency and latency of stress signals, since this was displayed only by a few puppies. Therefore, we decided to record the stress signals as a binomial factor, “Stress”, consisting of two categories: whether they showed any stress signal or none. All behaviors not included in the above categories were added to the category “Other” (see Table 1).

**Table 1:** Behaviors recorded in the problem-solving task

Categories	Behaviors	Definition
FRTI interactions	Tactile approach	Any behavior involving the puppy being in contact with the FRTI, e.g., rubbing, licking, pawing, scratching
	Gaze at the FRTI	The puppy from a stationary position gazing at the FRTI
	To head toward the FRTI	The puppy goes toward the FRTI from any position of the room. The recording of the behavior starts when the puppy focused on the FRTI
Social interest	Gaze at the owner	From a stationary position, puppy turns/lifts its head toward the handler, without approach
	Gaze at the stranger	From a stationary position, puppy turns/lifts its head toward the stranger, without approach
	Interaction with the owner	The puppy establishes physical contact with the owner, e.g., rubbing, nosing, licking, pawing a hand or leg or jumping up
	Interaction with the stranger	The puppy establishes physical contact with the stranger, e.g., rubbing, nosing, licking, pawing a hand or leg or jumping up
	To head toward the owner	The puppy goes to the owner from anywhere in the room. The recording of the behavior starts when the puppy focuses on the owner
	To head toward the stranger	The puppy goes to the stranger from anywhere in the room. The recording of the behavior starts when the puppy focuses on the stranger
Stress	Stress signals	Includes all behaviors indicating stress (i.e., yawning, scratching, shaking, licking lips, barking, locomotion without a clear target)
Other	Mixed	Includes all behaviors not included in the above categories (i.e., moving or gazing toward other targets different from the FRTI or people, such as the door, the bowl, or the walls); exploring (both visual and olfactory); passivity

*FRTI* food retrieval task instrument

The behaviors were analyzed by the Solomon Coder^®^ beta 16.06.26 (ELTE TTK, Hungary). The data were coded by an expert researcher, while a second independent researcher randomly coded only 16 videos (about 21%) of the total sample to test inter-observer reliability. The level of agreement ranged from 93 to 99% as a function of the item examined.

In a preliminary analysis, we first used the Mann–Whitney *U* test to explore statistical differences between the sexes on the Performance Index, Speed, and the latencies and frequencies of the Social Interest and the FRTI Interactions. Then, we applied two Generalized Linear Models (GzLM). In the first model, Speed (i.e., the timing required to solve the first task) was set as the response variable, and Sex and Stress were the explanatory factors. Speed constituted latency was tested in a GzLM model using the Social Interest and FRTI Interactions latencies as covariates. We also inserted Other and Age as covariates for a more comprehensive model. In the second model, the Performance Index was the response variable. Sex and Stress were again the explanatory factors. Being the Performance Index measured as a frequency, we coherently used the frequencies of the covariates (i.e., Social Interest, FRTI Interactions, Age, and Other). The main effects of factors (i.e., Sex and Stress) and covariates and the first level of interaction of Sex with Stress and the covariates were tested. The Performance Index followed a classical Poisson distribution (One-Sample Kolmogorov–Smirnov Test: *z* = 0.64, *p* = 0.82), whereby the Poisson log-linear models of GzLM were chosen. The variable Speed was skewed, so a GzLM with Tweedie log link distribution was applied.

All analyses were carried out in SPSS (SPSS Statistics, version 24; IBM Corp., Armonk, NY, USA).

## Results

All puppies quickly interacted with task items on the FRTI (Mean ± SD: males: 0.16 ± 0.47; females: 0.34 ± 1.08). Three males (8%) and eight females (21%) were unsuccessful (i.e., zero tasks solved), while four male puppies (10%) and six females (16%) completed all solvable tasks (i.e., 5). Thirty-two females (84%) manifested Social Interest, and 28 males (72%) did so. Stress signals were recorded in 11 male cases (28%) and 10 female cases (26%).

**Fig. 2:**
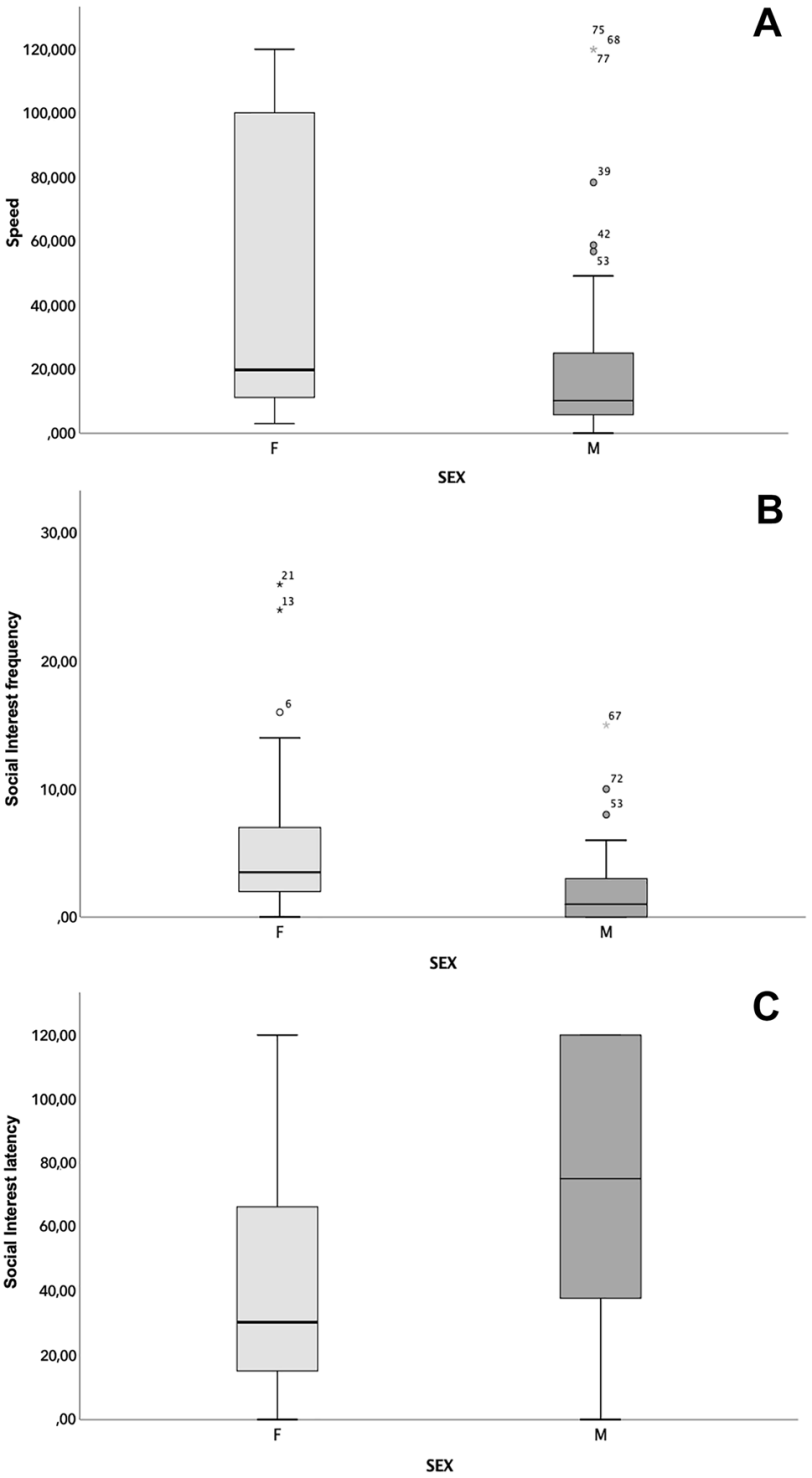
Graphics of significant sex differences as reported in Table 2. The box plots compare sex according to speed (**A**), social interest frequency (**B**), and social interest latency (**C**)

The Mann–Whitney *U* test revealed sex differences (Table 2) in Speed, with males resolving task items faster than females (Fig. 2A). Females exhibited more frequent (Fig. 2B) and faster (Fig. 2C) Social Interest. No significant sex differences were found for the Performance Index and the FRTI Interactions (Table 2).

**Table 2:** Mann–Whitney *U* test

		F_median [q1; q3]	M_median [q1; q3]	*U*	*Z*	*p*
Performance index	n°	2 [1; 3]	2 [1; 4]	617,000	− 1.285	0.199
**Speed**	**Latency**	19,8 [10,8; 104,2]	10,2 [5,8; 27,6]	484,500	− 2.617	**0.009**
**Social interest**	**Frequency**	3,5 [2; 7,75]	1 [0; 3]	479,500	− 2.691	**0.007**
	**Latency**	30,4 [13,5; 70,95]	75,2 [37,6; 120]	468,000	− 2.797	**0.005**
FRTI interactions	Frequency	12 [9,75; 15]	11 [8; 14]	665,000	− 0.777	0.437
	Latency	0 [0; 0,2]	0 [0; 0]	649,000	− 1.292	0.196

Statistical parameters, median, and interquartile ranges according to Sex. In bold are significant differences

*FRTI* food retrieval task instrument

The GzLM with Speed set as the response variable showed that the full model’s likelihood ratio chi-square test significantly improved fit over a null (Omnibus Test: *χ*^2^ = 50.234, *p* < 0.001). The model reported a negative main effect of Sex (*β* = − 1.092; *χ*^2^ = 5.083; *p* = 0.024), with males showing a higher probability of solving the first task faster than females (reference category). There was also a negative main effect of the Social Interest latency (*β* = − 0.015; *χ*^2^ = 18.832; *p* < 0.001) (i.e., a decrease of Social Interest latency will increase the Speed) and positive interaction between male Sex and Stress (*β* = 0.889; *χ*^2^ = 4.405; *p* = 0.036) (i.e., the group of males showing stress had less probability of being faster in solving the task). No main effects of Stress or FRTI Interactions emerged, nor did any statistical interaction between Sex and the latencies of the covariates.

The GzLM with the Performance Index as the response variable, Sex, and Stress as explanatory factors, with the frequencies of Social Interest, FRTI Interactions, Other, and Age as covariates, produced a likelihood ratio Chi-square test indicating a significant improvement of the full model over a null (Omnibus Test: *χ*^2^ = 42.033, *p* < 0.001). There was no main effect for Sex or Stress on the Performance Index, but there was a main effect of both Social Interest (*β* = − 0.108; *χ*^2^ = 10.705; *p* = 0.001) and FRTI Interactions (*β* = 0.061; *χ*^2^ = 6.111; *p* = 0.013), which worked in opposite directions. Decreasing Social Interest and increasing FRTI Interaction frequencies increase the probability of performing better, irrespectively of Sex or Stress.

## Discussion

The current study highlighted differences in problem-solving strategies in male and female puppies. The Performance Index was the indicator of the efficiency in solving multiple tasks, with Speed as a measure of the problem-solving efficiency in a single task (Chow et al. 2016). Non-parametric tests showed that males were significantly quicker than females in solving the first task. We did not apply a statistical correction for multiple comparisons. However, the GzLM reported a higher probability for males to solve the first task item faster, thus making our outcome robust. Therefore, we have replicated the results of Duranton et al. (2015) with adult dogs in puppies. The Duranton et al.’s (2015) study suggested that the lower level of neophobia toward the FRTI favored males’ confidence with the task, which led to higher success. The finding that adult male dogs are bolder than females and females are more likely to be fearful or anxious than males is well known (Goddard and Beilharz 1982; Svartberg 2002; Starling et al. 2013; Salonen et al. 2020), which lends plausibility to Duranton and colleagues’ hypothesis. Neophobic responses to a task could be deduced from how the subject approaches the FRTI. For example, longer latencies and lower frequencies interacting with a new tool could indicate a higher neophobic tendency. However, no sex differences in the latencies in approaching the task emerged between male and female adult dogs (Duranton et al. 2015) and in our results for puppies.

Moreover, the frequencies of the interactions with the FRTI also failed to discriminate sexes in puppies. These results show no support for different neophobic responses in males and females, both in adult dogs and puppies. In children (Clyman et al. 1986), as well as in dogs (Merola et al. 2012, 2013), it has been shown that social interest increases in stressful situations, which could suggest the Social Interest of puppies as an indirect measure of the level of neophobia toward the FRTI. However, our statistical models, which included Speed and Performance Index as response variables, failed to show interactions between Sex and Social Interest. These do not allow us to support the idea that potentially different neophobic responses between sexes could explain the different outcomes of male and female puppies.

Stress appears to affect performance success in our experimental paradigm. Indeed, the increase in stress of male puppies is related to the timing to solve the task. Research findings on the effects of stress show contrasting findings. Some studies report that stress increases performance in males, and negatively affects females (Schoofs et al. 2013). However, our data with male puppies converge with the findings of a study that shows higher stress responses inducing a decline of performance in men (Luers et al. 2020). Nevertheless, our results for females (who appeared not affected by stress) are not in line with those of women who benefit from higher stress (Luers et al. 2020).

One factor explaining our study’s performance success was Social Interest, although without being qualified by sex. The faster and more frequently puppies displayed Social Interest, the worse their outcome in the case of both sexes. Although female puppies were more prone to social interactions with humans than males, this factor does not explain the higher probability of males solving the task faster. In a previous study in which puppies were tested in the impossible task paradigm, the authors found no sex differences in social interest, measured by the amount of gazing behavior toward humans (Passalacqua et al. 2011). On the other hand, our results agree with that of Lazarowski et al. (2021), who reported a lower tendency in social engagement in 11-month-old adolescent males, and with several reports in adults (D’Aniello et al. 2016; Duranton et al. 2016; Eken Asp et al. 2015; Foyer et al. 2013; Kubinyi et al. 2009; Mongillo et al. 2016; Persson et al. 2015). Therefore, female social susceptibility could be a trait emerging early on during ontogenesis.

## Conclusions

Our study highlights sex differences in a problem-solving task in dog puppies for the first time, thus highlighting that sexually dimorphic behavioral differences in problem-solving strategies develop early on during ontogenesis. Males are probably quicker than females to solve the first task, but there were no sex differences when considering the whole performance. Stress affected specifically males, negatively influencing their timing to solve the task. The other two factors examined, Social Interest and the FRTI Interactions, impacted the performances oppositely, but none of them specifically in terms of the sexes, despite female puppies appearing more socially oriented than males.

Sex is a dimorphic trait, since adult males and females have different roles and behavioral ecology. Puppies do not show different roles and behavioral ecology; thus, the intriguing question is why sex-based behavioral differences should already be present in puppies. One explanation could be that sex differences emerge very early as preparatory for adult life so that sex-specific tendencies could be exercised and reinforced during development.

## Supplementary Information

Below is the link to the electronic supplementary material.

Supplementary file1 (PDF 38 KB)

## Data Availability

All tables and graphical data obtained during this study are included in this published article. The datasets generated and/or analyzed during the current study are available from the corresponding author on request.
