# Supplementary material for: Puppies in the problem-solving paradigm: quick males and social females
Source: Anim Cogn. 2022 Nov 22;26(3):791–7. doi: 10.1007/s10071-022-01714-5 (PMC10066122; doi:10.1007/s10071-022-01714-5)
Supplement: Supplementary file 1 — Supplementary file1 (PDF 38 KB) [file 10071_2022_1714_MOESM1_ESM.pdf]

ID	Breed	Age	Sex
01_PSp	Golden retriever	4	F
02_PSp	Mix	4	F
03_PSp	Jack Russel	5	M
04_PSp	Setter	4	M
05_PSp	Mix	4	M
06_PSp	Mix	4	M
07_PSp	Setter	4	M
08_PSp	Labrador retriever	6	F
09_PSp	Mix	4	F
10_PSp	Mix	4	M
11_PSp	Setter	4	F
12_PSp	Dachshund	6	F
13_PSp	Dachshund	5	F
14_PSp	Golden retriever	6	M
15_PSp	Golden retriever	3	F
16_PSp	Rottweiler	4	M
17_PSp	Labrador retriever	6	F
18_PSp	Maltese	5	F
19_PSp	Mix	5	F
20_PSp	German Spitz	5	M
21_PSp	Labrador retriever	6	M
22_PSp	Mix	5	F
23_PSp	Mix	6	M
24_PSp	Mix	4	M
25_PSp	Mix	6	F
26_PSp	Labrador retriever	6	F
27_PSp	Border collie	4	F
28_PSp	Labrador retriever	6	F
29_PSp	Mix Chihuahua/Pincher	4	M
30_PSp	Labrador retriever	3	F
31_PSp	Mix	6	F
32_PSp	Mix	3	F
33_PSp	Labrador retriever	4	M
34_PSp	Mix	5	F
35_PSp	Irish setter	5	M
36_PSp	Irish setter	5	F
37_PSp	Mix	5	F
38_PSp	Labrador retriever	3	M
39_PSp	Mix Pit	4	M
40_PSp	Labrador retriever	3	M
41_PSp	Mix Maremmano/Golden	5	F
42_PSp	Cane corso	6	F
43_PSp	Pug	3	F
44_PSp	Border collie	5	F
45_PSp	Border collie	6	F
46_PSp	Cocker	6	F
47_PSp	Labrador retriever	6	F
48_PSp	Golden retriever	4	M
49_PSp	Bulldog francese	4	M
50_PSp	Mix	5	M

51_PSp	Golden retriever	5	M
52_PSp	Mix	4	F
53_PSp	Mix	5	F
54_PSp	Rhodesian ridgeback	3	F
55_PSp	Mix	6	M
56_PSp	Poodle	6	M
57_PSp	Mix	5	F
58_PSp	Golden retriever	6	M
59_PSp	Golden retriever	4	M
60_PSp	Mix	4	F
61_PSp	Golden retriever	6	M
62_PSp	Golden retriever	3	F
63_PSp	Labrador retriever	6	M
64_PSp	American Bully	5	M
65_PSp	Mix	5	M
66_PSp	Golden retriever	5	M
67_PSp	German Shepherd	6	M
68_PSp	Dachshund	6	M
69_PSp	Beagle	5	F
70_PSp	Labrador retriever	6	M
71_PSp	Labrador retriever	5	M
72_PSp	Mix	6	F
73_PSp	Labrador retriever	6	M
74_PSp	Dachshund	4	M
75_PSp	Belgian shepherd	3	M
76_PSp	Golden retriever	4	M
77_PSp	Labrador retriever	4	F
